# High-Sensitivity Troponin T: A Biomarker for Diuretic Response in Decompensated Heart Failure Patients

**DOI:** 10.1155/2014/269604

**Published:** 2014-08-28

**Authors:** João Pedro Ferreira, Mário Santos, Sofia Almeida, Irene Marques, Paulo Bettencourt, Henrique Carvalho

**Affiliations:** ^1^Internal Medicine Department, Centro Hospitalar do Porto, Largo Professor Abel Salazar, 4099-001 Porto, Portugal; ^2^Centro Hospitalar do Porto, Porto, Portugal; ^3^Climate Change Impacts, Adaptation and Mitigation Research Group (CC-IAM), Faculdade de Ciências, Universidade de Lisboa, 1749-016 Lisbon, Portugal; ^4^Centro Hospitalar de São João, Alameda Professor Hernani Monteiro, 4200-319 Porto, Portugal

## Abstract

*Background.* Patients presenting with acutely decompensated heart failure (ADHF) and positive circulating cardiac troponins were found to be a high-risk cohort. The advent of high-sensitive troponins resulted in a detection of positive troponins in a great proportion of heart failure patients. However, the pathophysiological significance of this phenomenon is not completely clear. *Objectives.* The aim of this study is to determine the early evolution and clinical significance of high-sensitivity troponin T (hsTnT) in ADHF. *Methods.* Retrospective, secondary analysis of a prospective study including 100 patients with ADHF. *Results.* Globally, high-sensitivity troponin T decreased from day 1 to day 3 (*P* = 0,039). However, in the subgroup of patients who remained decompensated no significant differences in hsTnT from day 1 to day 3 were observed (*P* = 0,955), whereas in successfully compensated patients a significant reduction in hsTnT levels was observed (*P* = 0,025). High-sensitivity troponin T decrease was correlated with NTproBNP reduction (*P* = 0,007). Patients with hsTnT increase had longer length of stay (*P* = 0,033). *Conclusions.* Episodes of ADHF are associated with transient increases in the blood levels of hsTnT that are reduced with effective acute episode treatment. The decrease in hsTnT can translate less myocardial damage along with favourable ADHF treatment.

## 1. Introduction

Patients presenting with acutely decompensated heart failure (ADHF) and positive circulating cardiac troponins were found to be a high-risk cohort, requiring greater use of hospital resources and having increased risk of in-hospital mortality [1]. Measurement of cardiac troponins in this setting adds important prognostic information and should be considered as part of an early assessment of risk [1, 2].

Detectable troponins, even in the absence of acute coronary syndrome, are associated with impaired hemodynamics, progressive decline in left ventricular systolic function, and shortened survival [3–5].

Recent improvements in the sensitivity of troponin assays added additional challenges in the interpretation of these biomarkers in heart failure (HF). The increasing sensitivity of more contemporary assays has resulted in the detection of circulating troponin in a progressively greater proportion of HF patients. This phenomenon has led to increasing uncertainty about the clinical interpretation of troponin data from contemporary assays, particularly in patients with ADHF, since a substantial proportion of these patients have elevations of circulating troponins [1, 6, 7].

The aim of this study is to determine the early evolution, associations, and correlations of high-sensitivity troponin T (hsTnT) in ADHF.

## 2. Methods

### 2.1. Study Design

We analysed a database from a previous conducted prospective, interventional trial that we performed [8]. In that study we enrolled 100 consecutive patients who presented in a Portuguese tertiary hospital with ADHF, between February 2012 and February 2013. They were assigned in a sequential 1 : 1 ratio to spironolactone plus standard ADHF therapy or standard ADHF therapy alone. Patients were eligible for enrollment if they presented with decompensation of chronic HF with symptoms leading to hospitalization. ADHF was diagnosed on the basis of the presence of history of chronic HF and at least one symptom (dyspnea, orthopnea, or edema) and one sign (rales, peripheral edema, ascites, or pulmonary vascular congestion on chest radiography). Exclusion criteria were chronic use of mineralocorticoid receptor antagonists (MRAs), cardiac surgery within 60 days of enrollment, cardiac mechanical support, cardiac resynchronization-therapy within the last 60 days, comorbid conditions with an expected survival of less than 6 months, acute MI at time of hospitalization, hemodynamically significant uncorrected primary cardiac valvular disease, patients requiring intravenous vasodilators or inotropic agents, supine systolic arterial blood pressure <90 mmHg, plasma creatinine (pCr) level >1,5 mg/dL, serum potassium level >5,0 mmol/L, hemoglobin (HgB) level <9 g/dL, and sepsis.

Institutional review board or ethics committee approval was obtained. All patients provided written informed consent to participate in the study.

### 2.2. Study Assessments

Patient's clinical assessment including physical examination was prospectively recorded daily by the same assistant physician.

Medications and respective dosages were prospectively recorded by the investigators according to the assistant physician prescriptions.

Blood and spot urine samples were collected in the first 24 hours (h) after admission (day 1) of the patient to the hospital. The day 3 samples were collected between 72 and 96 h of hospitalization. An assessment of biomarkers, including pCr, plasma urea (pUr), electrolytes, N-terminal probrain natriuretic peptide (NTproBNP), and hsTnT, was performed at a central core laboratory at day 1 and day 3. Clinical assessment and routine analyses were performed daily during hospital stay. Estimated glomerular filtration rate (eGFR) was determined using the chronic kidney disease epidemiology collaboration (CKD-EPI) equation [8]. All patients performed a transthoracic echocardiography within 72 hours upon admission. Ejection fraction (EF) was calculated according to biplane Simpson method.

High-sensitive troponin T was measured using COBAS Troponin T hs (highly sensitive) STAT (short turn-around time) (Roche Diagnostics). According to the manufacturer a positive hsTnT test was considered when the value was above the upper reference limit (99th percentile) of 0,014 ng/mL.

### 2.3. Variable Definitions

We studied hsTnT regarding the following covariates: comorbidities such as diabetes mellitus (DM), chronic obstructive pulmonary disease (COPD), and sleep apnea; body mass index (BMI); heart rate (HR); systolic blood pressure (SBP); atrial fibrillation (AF); HF etiology; echocardiographic parameters such as EF; furosemide dose, proportion of patients on angiotensin converting enzyme inhibitors (ACEi), beta-blockers (BB), and spironolactone; pCr, pUr, NTproBNP, sodium, and potassium; HgB and serum albumin.

In order to determine the differences in hsTnT concentration between patients with faster diuretic response and patients with slower diuretic response after 3 days of inpatient treatment, patients were considered faster diuretic responders if they had decreased intravenous (i.v.) furosemide dose or switched to oral furosemide in the first three days of in-hospital treatment. On the other hand, patients were considered to be slower diuretic responders if the assistant physician increased or maintained i.v. furosemide dosage after three days of in-hospital treatment.

### 2.4. Statistical Analysis

Normally distributed continuous variables are expressed as mean ± standard deviation (SD), and skewed distributions are presented as median (interquartile range (IQR)).

Categorical variables are expressed in absolute numbers (no.) and proportions (%).

Comparison between groups was performed using parametric, nonparametric tests, or chi-square tests, as appropriate. Significant association was defined by a probability (*P*) value ≤ 0,05.

The positively skewed distributions were log transformed for analysis.

Correlations of log hsTnT were first examined by single variable linear or logistic regression and presented as nonadjusted coefficient (NAC) and 95% confidence interval [_95%_CI]. Factors with a *P* value ≤ 0,05 by single variable regression analyses were included in a multivariable linear regression model, presented as adjusted coefficient (AC) [_95%_CI].

Statistical analysis was performed using SPSS software (version 19, Chicago, IL, USA).

## 3. Results

### 3.1. Baseline Characteristics and Early Changes

Mean ± SD age of the 100 patients admitted due to ADHF was 76 ± 10,9 years. Thirty-nine (39%) patients were male; 50 patients had documented ischemic heart disease (IHD); 59 had AF; and mean ± SD EF (%) was 43,46 ± 11,73 (Table 1). All patients were admitted in New York Heart Association (NYHA) class IV. Patient characteristics, medications, and comparison of lab results between admission day (day 1) and the third day of inpatient treatment are shown in Table 1.

**Figure 1:**
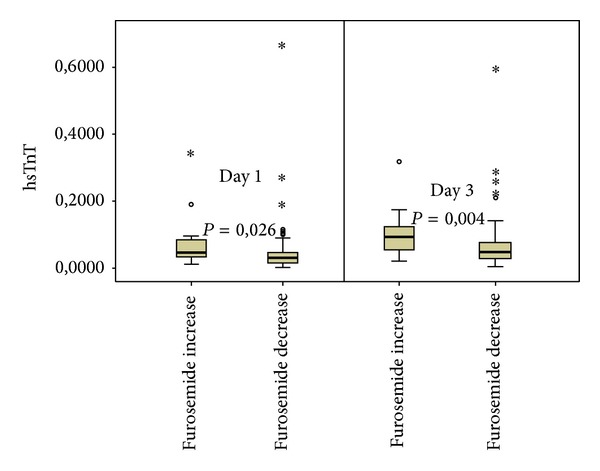
Differences in hsTnT between faster diuretic responders and slower diuretic responders at day 1 and day 3. hsTnT: high-sensitivity troponin T (ng/mL).

Globally, high-sensitivity troponin T was likely to decrease from day 1 to day 3 (median [IQR], 0,033 [0,019–0,050] versus 0,030 [0,018–0,051], *P* = 0,039) (Table 1). However, in the subgroup of patients considered to have slower diuretic response no significant differences in hsTnT from day 1 to day 3 were observed (median [IQR], from 0,046 [0,033–0,087] to 0,055 [0,032–0,072], *P* = 0,955), whereas in the group of patients considered to have a faster diuretic response a significant reduction in hsTnT levels was observed (median [IQR], from 0,032 [0,017–0,048] to 0,028 [0,017–0,045], *P* = 0,025) (Table 2 and Figure 1). The hsTnT variation did not differ between groups (median [IQR], −0,0005 [−0,043 to 0,004] versus −0,0010 [−0,020 to 0,002], *P* = 0,51) (Table 2). The majority of patients with negative hsTnT at day 1 remained negative at day 3 (76,9%). On the other hand only a small proportion (3,1%) of patients with positive hsTnT at day 1 turned negative at day 3 (Table 3).

### 3.2. High-Sensitivity Troponin T Correlations

**Figure 2:**
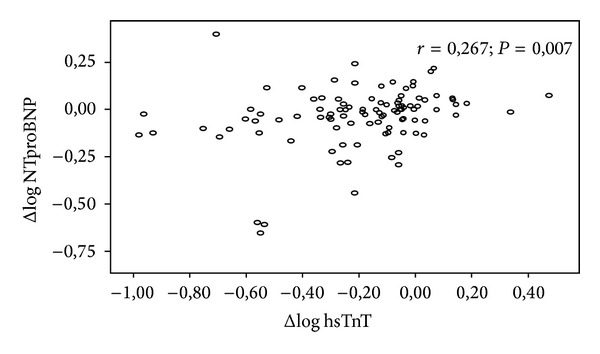
Correlation between ΔLog hsTnT and ΔLog NTproBNP. hsTnT: high-sensitivity troponin T (ng/mL); NTproBNP: N-terminal probrain natriuretic peptide (pg/mL); Δ: changes between day 3 and day 1 (day 3–day 1).

Bivariate analysis of hsTnT at day 1 found positive correlations with day 1 Log NTproBNP (NAC [_95%_CI], 0,481 [0,267 to 0,574], *P* < 0,001), pUr (NAC [_95%_CI], 0,309 [0,002 to 0,009], *P* = 0,002), and Log pCr (NAC, [_95%_CI], 0,345 [0,500 to 1,704], *P* < 0,001). A negative correlation was found with Log eGFR (NAC [_95%_CI], −0,275 (−1,231 to −0,216), *P* = 0,006). Day 3 hsTnT was also positively correlated with day 3 Log NTproBNP (NAC [_95%_CI], 0,486 [0,218 to 0,464], *P* < 0,001) and Log pCr (NAC, [_95%_CI], 0,439 [0,630 to 1,503], *P* < 0,001) and negatively correlated with Log eGFR (NAC [_95%_CI], −0,399 (−1,232 to −0,455), *P* = 0,006) (Table 4). High-sensitivity troponin T decrease was correlated with NTproBNP reduction (NAC [_95%_CI], 0,267 [0,044 to 0,276], *P* = 0,007) (Table 4 and Figure 2). By multivariate analysis, hsTnT correlated with NTproBNP at day 1 and day 3 (AC [_95%_CI], 0,400 [0,185 to 0,513], *P* < 0,001, and 0,381 [0,146 to 0,389], *P* < 0,001, resp.) (Table 4).

### 3.3. Determinants of hsTnT Change

High-sensitivity troponin T was transformed according to the pattern of change (decrease or increase) during the first 3 days of treatment (Table 5).

Patients with hsTnT increase had lower NTproBNP decrease (median [IQR], −1167 [−2337 to −367] versus −379 [−1273 to 319,5], *P* = 0,003), had longer length of stay (median [IQR], 8 [6 to 11] versus 9 [7 to 12], *P* = 0,033), and had higher proportion of AF (49,2% versus 75,7%, *P* = 0,009). Diuretic dosages, other HF medications, renal function, and length of stay did no differ between groups (Table 5).

## 4. Discussion

The major finding of this study is that episodes of ADHF are associated with transient increases in the blood levels of hsTnT that are reduced with acute episode effective treatment. This statement is corroborated by the higher levels of hsTnT in patients who maintained or increased i.v. furosemide dose after 3 days of hospitalization, by a decrease in hsTnT levels in patients with faster response to diuretic therapy, by the correlation between troponin T decrease and NTproBNP reduction, and by the longer length of stay and lower decrease in NTproBNP levels in the group of patients who had increase in hsTnT from day 1 to day 3.

Improvements in analytical sensitivity have transformed circulating troponin from a biomarker that was only detectable in a minority of patients to one that is detectable in the vast majority of patients with HF [1]. The high sensitivity of the test can detect very small changes in the circulating troponin levels [1, 7], providing a potential explanation for the high proportion of patients who remained above the 99th percentile after 3 days of treatment.

In our study over 80% of the patients had hsTnT levels above the 99th percentile; this prevalence of detectable hsTnT was higher than in previously published reports [1–3, 6, 9, 10]. The most likely explanation for this finding is the type of tests, assay platforms, and the cutoff limits used in those studies. For example, the acute decompensated heart failure national registry (ADHERE) study used a higher cutoff limit of 0,1 ng/mL and they did not control the assay platform [1], and in the enhanced feedback for effective cardiac treatment (EFFECT) study the cutoff limit used was 0,5 ng/mL^3^. However, in the another study by Metra et al. [6] the used cutoff was 0,01 ng/mL where levels above this value were considered abnormal. In that study, 51 (48%) of the 107 patients discharged alive from the hospital had detectable troponin in at least one measurement. Despite the differences in the type of test and assay platform, the cutoff limit was similar to the cutoff used in our study. One possible explanation for this discrepancy is the mean ± SD age of the patients included in the present study. Our patients are older than patients included in the study by Metra et al. (76 ± 10,9 versus 66 ± 13 years, resp.). Troponin levels are likely to have a Gaussian or near Gaussian distribution, with higher levels found in older age groups [11].

Elevations in baseline troponin levels were demonstrated to be independent predictors of events during the acute hospitalization (worsening or persistent HF, death, and increased length of stay) and also independent predictors of postdischarge outcomes [3, 6, 9, 12–14]. In our study, an increase in hsTnT levels was also associated with longer length of stay consistent with the previous cited reports.

Changes in troponin status during initial treatment for ADHF have been proposed as potentially important targets for drug development [15]. In the biomarker analysis from the Relaxin in acute heart failure (RELAX-AHF) development program [16], changes in markers of cardiac (hsTnT), renal (pCr and cystatin-C), and hepatic (aspartate transaminase and alanine transaminase) damage and of decongestion (NTproBNP) at day 2 improved with Serelaxin administration. These findings were consistent with the prevention of organ damage and faster decongestion. Our study also showed a reduction in hsTnT levels in the first days of HF treatment in patients who were able to reduce i.v. furosemide dose or switch it to oral route and in patients with higher reduction in natriuretic peptides, possibly traducing less myocardial damage in patients with more favourable therapeutic response, that is, faster decongestion. This finding provides additional data supporting the use of troponin as a biomarker for ADHF severity and therefore a potential therapeutic target. In addition, NTproBNP and hsTnT are independent markers of increased mortality risk in HF [6, 14, 17, 18] and natriuretic peptides have shown to correlate with changes in ventricular wall stress, being inversely related to the severity of left ventricular dysfunction [17, 19–21]. A decline in NTproBNP plasma levels during the initial hospitalisation was observed in our study, a finding consistent with previous reports [22–25]. Furthermore, this study demonstrates that patients with hsTnT decrease have a more pronounced NTproBNP reduction, and a weak but positive correlation between hsTnT and NTproBNP was found. Despite the weak correlation between hsTnT and NTproBNP, these results may suggest that congestion and ventricular wall stress relief can be translated into natriuretic peptide and hsTnT reduction. However, the different pathophysiological mechanisms targeted by these biomarkers may explain the weak correlation between them described in this study. Nevertheless, this finding was not observed in other studies involving patients with heterogeneous HF presentations [6, 26].

The ADHF episodes are associated with increased mechanical strain on the heart, activation of neurohormonal systems, and increased and oxidative stress [25]. These stimuli are known to mediate myocardial injury, accelerating myocyte loss [25]. Troponin T is highly specific for cardiac myocytes, but circulating levels may also be elevated due to renal insufficiency. However, this mechanism does not seem to underlie our observations, since hsTnT is positively correlated with pCr and negatively correlated with eGFR at day 1 and day 3, but the changes in hsTnT during treatment are not correlated with changes in renal function. Thus, patients with impaired renal function are likely to have higher hsTnT levels, but hsTnT reduction is independent of renal function changes. Thus, we believe that the elevation in hsTnT reflects increased release from the myocardium and, thus, may indicate myocyte injury and/or death.

In the group of patients with hsTnT increase a higher proportion of patients with AF were observed. These findings are consistent with previous larger trials, in which a positive hsTnT was detected in almost all patients with AF, with hsTnT levels carrying strong and independent prognostic information with a gradual increase in the risk of stroke, cardiac, and total death [27]. Our study was underpowered for major cardiovascular events and death, but a longer length of stay was observed in patients with hsTnT increase as discussed above.

## 5. Limitations

Our study has several limitations that need to be considered. It was a single-centre investigation of a small sample, which limits our inferential analysis. The decision to withdraw diuretic therapy was based on subjective assessment of congestive signs and symptoms, so we cannot rule out the interobserver variability. However, in real-life clinical practice, the decision to step down diuretic therapy is also based on subjective clinical evaluation. Our study protocol defined that the first blood sample would be collected in the first 24 h, so at the time of venous blood sampling patients could have been treated already with diuretics. Although we are not comparing diuretic-naïve patients at day 1 measurements, the overall effect of this bias would be an underestimated difference between day 1 and day 3, which does not significantly affect the internal validity of our study conclusions. Finally, the external validity of our conclusions is limited to normohypertensive and fluid overloaded HF patients with normal or mildly impaired renal function, since all these factors were considered inclusion criteria. On the other hand, our conclusions can be reproducible in this set of patients widely common in clinical practice.

## 6. Conclusions

Episodes of ADHF are associated with transient increases in the blood levels of hsTnT that are reduced with effective acute episode treatment. The decrease in hsTnT and NTproBNP can translate ventricular wall stress relief and less myocardial damage along with favourable ADHF treatment. Further studies are needed to examine the value of combining necrosis markers and natriuretic peptides in the clinical management of ADHF patients.

## Figures and Tables

**Table 1:** Population characteristics and comparison of clinical variables, laboratory results, and medications between admission day (day 1) and day 3.

Age (yrs)	76,0 ± 10,88		
Male sex—%	39		
Diabetes mellitus—%	45		
Glycated HgB (%)	7,02 ± 0,96		
COPD—%	17		
Dementia—%	12		
Sleep apnea—%	18		
Noninvasive ventilation—%	17		
Ischemic heart disease—%	50		
Atrial fibrillation—%	59		
LV ejection fraction (%)	43,46 ± 11,73		
LV ejection fraction ≥40%—%	68		

	Day 1	Day 3	*P* value

Body mass index (Kg/m^2^)	29,44 ± 6,17	28,35 ± 6,23	**<0,001**
Heart rate (bpm)	93,65 ± 24,35	76,41 ± 11,96	**<0,001**
SBP (mmHg)	139,79 ± 25,86	121,97 ± 16,2	**<0,001**
Plasma creatinine (mg/dL)	1,04 [0,89–1,31]	1,06 [0,85–1,40]	0,082∗
eGFR (mL/min/1,73 m^2^)	58,0 [44,0–72,0]	58,0 [39,25–72,75]	0,171∗
Plasma urea (mg/dL)	55,21 ± 20,84	62,3 ± 25,47	**0,001**
Serum potassium (mmol/L)	4,03 ± 0,51	4,04 ± 0,54	0,95
Serum sodium (mmol/L)	140,54 ± 4,38	140,68 ± 3,95	0,72
Hemoglobin (g/dL)	12,43 ± 2,07	—	—
Albumin (mg/dL)	3,68 ± 0,40	—	—
NTproBNP (pg/mL)	2750 [1672–6032]	1835 [902–3837]	**<0,001** ∗
hsTnT (ng/mL)	0,033 [0,019–0,050]	0,030 [0,018–0,051]	**0,039** ∗
IV furosemide—%	100	37	**<0,001** ∗∗
IV furosemide dose (mg/d)	75,80 ± 21,52	67,57 ± 25,54	**0,001**
Oral furosemide—%	0	63	—
Oral furosemide dose (mg/d)	0	74,6 ± 28,1	—
Furosemide dose reduction or oral route—%	—	84	—
ACEi—%	44	61	**<0,001** ∗∗
Ramipril Eq. dose (mg/d)	3,15 ± 2,04	3,36 ± 2,14	0,474
Beta-blocker—%	37	57	**<0,001** ∗∗
Bisoprolol Eq. dose (mg/d)	3,01 ± 1,08	2,96 ± 1,89	0,474
Spironolactone—%	50	50	1∗∗
Spironolactone dose (mg/d)	94,50 ± 23,31	62,74 ± 24,33	**<0,001**

Continuous variables are presented as mean value ± standard deviation [SD], *P* value or median [interquartile range (IQR)], *P* value. Categorical variables are presented as % of total (100 patients), *P* value.

∗Nonparametric paired sample test; ∗∗chi-square test.

COPD: chronic obstructive pulmonary disease; LV: left ventricular; eGFR: estimated glomerular filtration rate; NTproBNP: N-terminal probrain natriuretic peptide; hsTnT: high sensitivity troponin T; IV: intravenous; ACEi: angiotensin converting enzyme inhibitors.

**Table 2:** Comparison of TnT levels between patients who responded to diuretic therapy versus patients who needed to increase diuretic dose.

	Furosemide maintenance or increase (*n* = 16)	Furosemide decrease or oral administration (*n* = 84)	*P* value Between groups
hsTnT (ng/mL)			
Day 1	0,046 [0,033 to 0,087]	0,032 [0,017 to 0,048]	**0,026** ∗
Day 3	0,055 [0,032 to 0,072]	0,028 [0,017 to 0,045]	**0,004** ∗
ΔhsTnT	−0,0005 [−0,043 to 0,004]	−0,0010 [−0,020 to 0,002]	0,51∗
	*P* value within group	*P* value within group	
	0,955∗	**0,025** ∗	

Continuous variables are presented as median [interquartile range (IQR)], *P* value. ∗Nonparametric test.

hsTnT: high-sensitivity troponin T.

**Table 3:** Comparison of hsTnT values below (negative) and above (positive) the 99th percentile (≥0,014 ng/mL).

		Day 1		Total	*P* value
		Negative hsTnT—no. (%)	Positive hsTnT—no. (%)		
Day 3	Negative hsTnT—no. (%)	10 (76,9)	3 (3,4)	13 (13)	**<0,001** ∗∗
	Positive hsTnT—no. (%)	3 (23,1)	84 (96,6)	87 (87)	**<0,001** ∗∗
	Total	*13 (13) *	*87 (87) *	*100 *	

**Chi-square test. Legend: hsTnT: high-sensitivity troponin T.

**Table 4:** Associations with log⁡hsTnT at day 1, day 3 and changes between day 1 and day 3 (Δ).

	Nonadjusted coefficient for log⁡hsTnT	_ 95%_CI	*P* value	Adjusted coefficient for log⁡hsTnT	_ 95%_CI	*P* Value
Age	0,119	−0,001 to 0,005	0,240			
Male sex	−0,066	−0,086 to 0,043	0,515			
DM	0,095	−0,033 to 0,093	0,349			
HgBA1c	0,058	−0,046 to 0,067	0,707			
LVEF	0,089	−0,001 to 0,004	0,376			
Ischemic HF	−0,078	−0,087 to 0,038	0,442			
Beta-blocker	−0,004	−0,065 to 0,062	0,969			
ACEi	0,023	−0,057 to 0,072	0,820			
Spironolactone	−0,116	−0,099 to 0,026	0,251			
BMI						
Day 1	−0,205	−0,025 to 0,000	0,042			
Day 3	−0,087	−0,016 to 0,006	0,391			
ΔBMI	−0,008	−0,019 to 0,018	0,937			
HR						
Day 1	0,063	−0,002 to 0,004	0,533			
Day 3	−0,078	−0,008 to 0,004	0,438			
ΔHR	0,013	−0,001 to 0,001	0,899			
SBP						
Day 1	0,102	−0,001 to 0,004	0,314			
Day 3	0,098	−0,002 to 0,006	0,333			
ΔSBP	0,207	0,000 to 0,003	0,039			
log⁡NTproBNP						
Day 1	0,481	0,267 to 0,574	**<0,001**	0,400	0,185 to 0,513	**<0,001**
Day 3	0,486	0,218 to 0,464	**<0,001**	0,381	0,146 to 0,389	**<0,001**
Δlog⁡NTproBNP	0,267	0,044 to 0,276	**0,007**	—	—	—
log⁡Albuminuria						
Day 1	0,131	−0,035 to 0,172	0,193			
Day 3	0,220	0,012 to 0,203	**0,028**	0,088	−0,041 to 0,128	0,311
Δlog⁡Albuminuria	0,099	−0,038 to 0,113	0,325			
log⁡eGFR						
Day 1	−0,275	−1,231 to −0,216	**0,006**	0,165	−0,503 to 1,372	0,360
Day 3	−0,399	−1,232 to −0,455	**<0,001**	0,034	−0,812 to 0,957	0,870
Δlog⁡eGFR	0,068	0,203 to 0,413	0,502			
log⁡pCr						
Day 1	0,345	0,500 to 1,704	**<0,001**	0,270	−0,224 to 1,951	0,118
Day 3	0,439	0,630 to 1,503	**<0,001**	0,256	−0,393 to 1,641	0,226
Δlog⁡pCr	−0,040	−0,443 to 0,297	0,696			
pUrea						
Day 1	0,309	0,002 to 0,009	**0,002**	0,116	−0,002 to 0,007	0,342
Day 3	0,382	0,003 to 0,008	**<0,001**	0,121	−0,002 to 0,005	0,335
ΔpUrea	−0,172	−0,003 to 0,000	0,087			
Albumin at day 1	−0,049	−0,099 to 0,060	0,626			
Hemoglobin at day 1	0,076	−0,009 to 0,021	0,451			

Day 1 values are compared with day 1 hsTnT; day 3 values are compared with day 3 hsTnT; Δ, age, sex, DM, HgBA1c, LVEF, ischemic HF, and medications are compared with changes (Δ) in hsTnT between day 1 and day 3 (day 3–day 1).

DM: diabetes mellitus; HgBA1c: glycated hemoglobin; LVEF: left ventricular ejection fraction; HF: heart failure; ACEi: angiotensin converting enzyme inhibitors; BMI: body mass index; HR: heart rate; SBP: systolic blood pressure; NTproBNP: N-terminal probrain natriuretic peptide; hsTnT: high sensitivity troponin T; eGFR: estimated glomerular filtration rate; pCr: plasma creatinine; pUrea: plasma urea; Δ: changes between day 3 and day 1 (day 3–day 1).

**Table 5:** Determinants of hsTnT dichotomic changes.

	hsTnT		*P* value
	Decrease (*n* = 63)	Increase (*n* = 37)	
Age (years)	75,94 ± 11,92	76,11 ± 8,97	0,940
Male sex—no. (%)	22 (34,9)	17 (45,9)	0,275∗∗
DM—no. (%)	24 (38,1)	21 (56,8)	0,070∗∗
HGA1c (%)	6,93 ± 0,94	7,13 ± 1,00	0,475
Sleep apnea—no. (%)	7 (36,8)	11 (44)	0,632∗∗
NIV—no. (%)	10 (15,9)	7 (18,9)	0,695∗∗
IHD—no. (%)	32 (50,8)	18 (48,6)	0,836∗∗
AF—no. (%)	31 (49,2)	28 (75,7)	**0,009** ∗∗
LVEF (%)	43,37 ± 12,68	43,62 ± 10,08	0,917
LVEF ≥40%—no. (%)	41 (65,1)	26 (70,3)	0,594∗∗
HgB (g/dL)	12,32 ± 1,95	12,62 ± 2,28	0,478
Albumin (mg/dL)	3,68 ± 0,41	3,67 ± 0,39	0,924
ΔBMI (Kg/m^2^)	−1,08 ± 1,70	−1,10 ± 1,76	0,964
ΔHR (bpm)	−17,05 ± 20,71	−17,57 ± 29,15	0,917
ΔSBP (mmHg)	−18,56 ± 23,32	−16,57 ± 27,63	0,702
ΔpCr (mg/dL)	0,03 [−0,1 to 0,18]	0,02 [−0,06 to 0,11]	0,803∗
ΔeGFR (mL/min/1,73 m^2^)	−2,0 [−9,0 to 7,0]	−1,0 [−11,0 to 6,0]	0,937∗
ΔpUrea (mg/dL)	7,40 ± 20,59	6,59 ± 20,65	0,851
ΔNTproBNP (pg/mL)	−1167 [−2337 to −367]	−379 [−1273 to 319,5]	**0,003** ∗
ΔhsTnT (ng/mL)	−0,004 [−0,014 to −0,001]	0,004 [0,002 to 0,009]	**<0,001** ∗
ΔAlbuminuria (mg/g)	−6,10 [−38,50 to 2,40]	−23,70 [−90,75 to 11,05]	0,337∗
IV furosemide at day 1 (mg)	78,83 ± 21,61	74,05 ± 21,53	0,537
IV furosemide dose			
Maintenance or increase at day 3—no. (%)	9 (14,3)	7 (18,9)	0,542∗∗
ACEi—no. (%)	30 (47,6)	14 (37,8)	0,341∗∗
Beta-blocker—no (%)	22 (34,9)	15 (40,5)	0,574∗∗
Spironolactone—no. (%)	30 (47,6)	20 (54,1)	0,534∗∗
Length of stay (days)	8,0 [6,0 to 11,0]	9,0 [7,0 to 12,0]	**0,033** ∗

Continuous variables are presented as mean value ± standard deviation [SD], *P* value or median [interquartile range (IQR)], *P* value. Categorical variables are presented as absolute number (%), *P* value.

∗Nonparametric paired sample test; ∗∗chi-square test.

DM: diabetes mellitus; HgBA1c: glycated hemoglobin; NIV: noninvasive ventilation; IHD: ischemic heart disease; AF: atrial fibrillation; HgB: hemoglobin; BMI: body mass index; HR: heart rate; SBP: systolic blood pressure; eGFR: estimated glomerular filtration rate; pCr: plasma creatinine; pUrea: plasma urea; NTproBNP: N-terminal probrain natriuretic peptide; hsTnT: high sensitivity troponin T; IV: intravenous; ACEi: angiotensin converting enzyme inhibitors; Δ: changes between day 3 and day 1 (day 3–day 1).
